# 

**Published:** 1867-08

**Authors:** 


### CONTENTS FOR AUGUST.

ORIGINAL CONTRIBUTIONS.

Selections from Surgical Notes. By Moses Gunn, M.D., Prof. Surgery, Rush Medical College, ---- 353
Contagion of Cholera. By R. L. Rea, M.D., Chicago, ---- 355
Enlargement of the Colon. By Wm. Lewitt, M.D., Demonstrator of Anatomy in Rush Medical College, ---- 359
Rabies. By R. P. Hunt, M.D., Chicago, ---- 361
Retarded Congenital Syphilis.—Syphilitic Caries. By H. Durham, M.D., LaSalle, Ill., ---- 362
Progressive Paralysis of the Insane, ---- 365
On the Treatment of Asthma. By W. Anderson, M.D., Pontiac, Ill., ---- 367
Venomous Bites. By James T. Newman, M.D., Chicago, ---- 368
Electro-Magnetism as a means in the Treatment of Disease. By C. F. Hart, M.D., Chicago, ---- 371
A New Form of Suture. By D. Prince, Jacksonville, Ill., ---- 373
" " By T. G. Comstock, M.D., Attending Physician St. Louis Good Samaritan Hospital, ---- 374
Gelseminum. By Jonathan W. Brooks, Chicago, ---- 375
The Endoscope, and its Application to the Diagnosis and Treatment of Urinary Affections. By A. J. Desormeaux, M.D., Translated by R. P. Hunt, M.D. Continued from Page 352 ---- 385

EDITORIAL.

Supplement, ---- 377
Erratum, ---- 377
Contagiousness of Cholera, ---- 377
Medical Societies, ---- 377
Rush Medical College, ---- 377
The Endoscope, ---- 378
University of Wisconsin, ---- 378
Canada, ---- 379
Alumni Meeting, ---- 380
A Chapter about the Books, ---- 381
Death of Dr. Hunt, ---- 384
Obituary of Dr. George K. Amerman, ---- 384
Receipts Acknowledged, ---- 384

SUPPLEMENT.

Laying the Corner-Stone of Rush Medical College, ---- 401

